# Fabrication routes for one-dimensional nanostructures via block copolymers

**DOI:** 10.1186/s40580-017-0106-1

**Published:** 2017-05-10

**Authors:** Maithri Tharmavaram, Deepak Rawtani, Gaurav Pandey

**Affiliations:** Institute of Research & Development, Gujarat Forensic Sciences University, Sector 18-A, Near Police Bhavan, Gandhinagar, Gujarat 382007 India

**Keywords:** Block copolymer, Nanowire, Nanorod, Nanofiber, Nanotechnology

## Abstract

Nanotechnology is the field which deals with fabrication of materials with dimensions in the nanometer range by manipulating atoms and molecules. Various synthesis routes exist for the one, two and three dimensional nanostructures. Recent advancements in nanotechnology have enabled the usage of block copolymers for the synthesis of such nanostructures. Block copolymers are versatile polymers with unique properties and come in many types and shapes. Their properties are highly dependent on the blocks of the copolymers, thus allowing easy tunability of its properties. This review briefly focusses on the use of block copolymers for synthesizing one-dimensional nanostructures especially nanowires, nanorods, nanoribbons and nanofibers. Template based, lithographic, and solution based approaches are common approaches in the synthesis of nanowires, nanorods, nanoribbons, and nanofibers. Synthesis of metal, metal oxides, metal oxalates, polymer, and graphene one dimensional nanostructures using block copolymers have been discussed as well.

## Background

Nanotechnology is a relatively new field which involves control and exploitation of structures with sizes in the nanometer range. Goals in this field include fabrication of materials with properties that are novel and improved and are likely to impact different fields [1]. Nanotechnology can be applied in fields like electronics, environmental science, food safety, biotechnology, medicine, and cosmetic industry [2–7]. Such wide arrayed applications call for the development of nanostructures with well-defined size, form, and crystal structure.

**Fig. 1:**
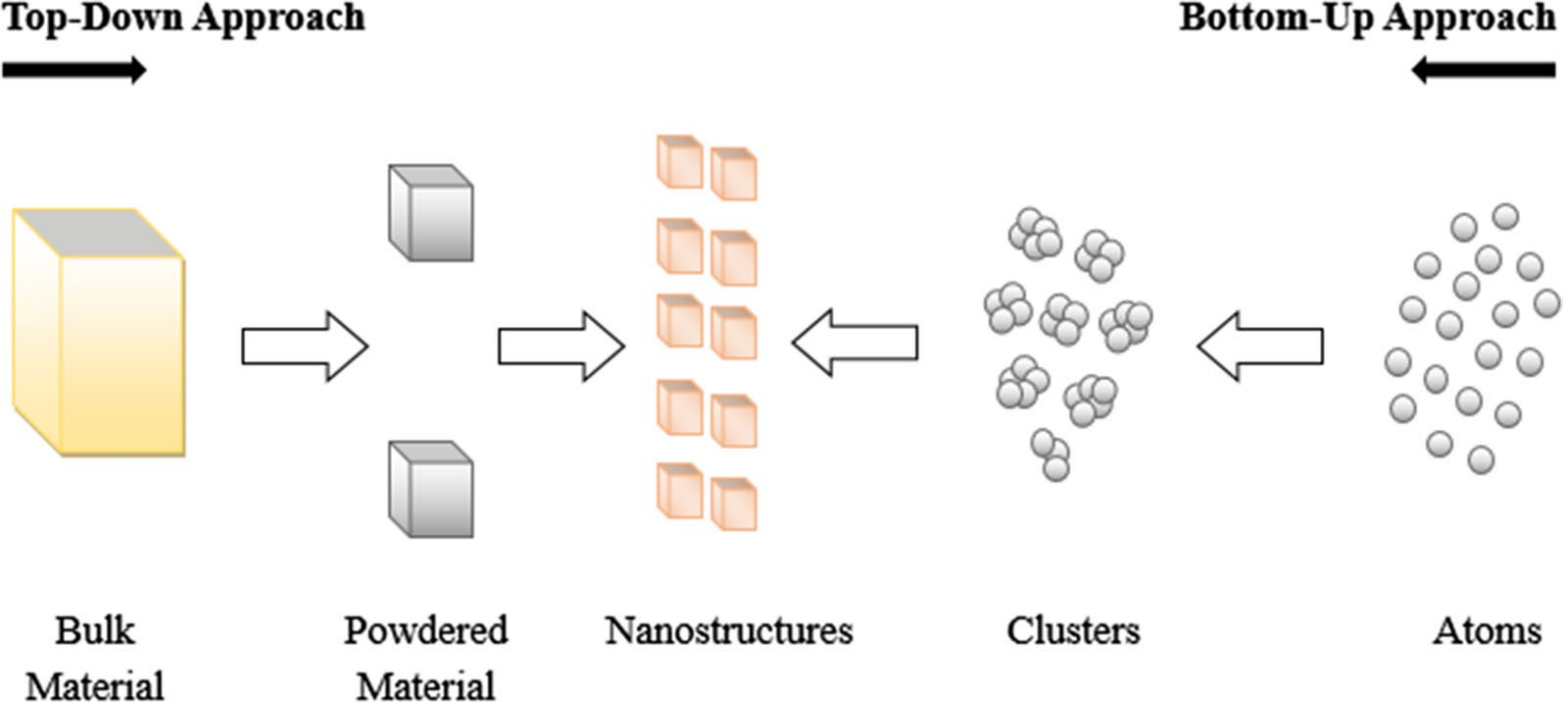
Approaches for synthesis of nanostructures [2]

Two main approaches exist for the synthesis of 1, 2 and 3-dimensional nanostructures such as nanowires, nanotubes, and nano-masks. These are top-down and bottom-up approach (Fig. 1). Top-down approach involves the breaking down of a bulk molecule into its smaller counterparts through rigorous mechanical processes. Bottom-up approach involves subjecting a precursor molecule to different chemical processes thus forming different nanostructures. Size control and contamination are some of the disadvantages of a top-down approach. Bottom-up approach minimizes some of these limitations. Tools like Atomic Force Microscopy (AFM), Scanning Electron Microscopy (SEM), Transmission Electron Microscopy (TEM), Near-field Scanning Optical Microscopy (SNOM), Scanning Probe Microscopy (SPM), X-Ray Diffraction (XRD), and Energy Dispersive X-Ray Spectroscopy (EDX) are some of the techniques generally used for this purpose.

One-dimensional nanostructures like nanowires, nanorods, nanoribbons, and nanofibers are providing a good system to investigate the dependence of thermal and electrical transport or mechanical properties on dimensionality and quantum confinement. They also play an important role as both functional units and interconnects in fabricating electronic, optoelectronic, electromechanical, and electrochemical device in the nanoscale dimension [8]. These nanostructures have been generally synthesized through techniques like chemical vapor deposition, VLS method, lithography, template based and solution based approaches.

Block copolymers are polymer alloys that have rubber-like behavior under normal conditions and can be easily molded at higher temperatures. This is due to the physical crosslinks provided by the glassy domains. Based on the molecular weight, the strength of interaction between the blocks, and segment size, the block copolymers can self-assemble to form nanostructures with specific domain spacing [9]. These properties along with their structure adaptability enable block copolymers to be used in the synthesis of nanostructures, especially one-dimensional nanostructures like nanowires, nanorods nanoribbons, and nanofibers [10].

## Block copolymers

### Types

Block copolymers have two or more polymer blocks arranged in a specific manner. These blocks are covalently bound together, chemically distinct, and immiscible. Such a structure allows the mechanical, electrical, optical and mass transport behavior of block copolymers to be easily tuned just by tweaking its molecular construction [11].

**Fig. 2:**
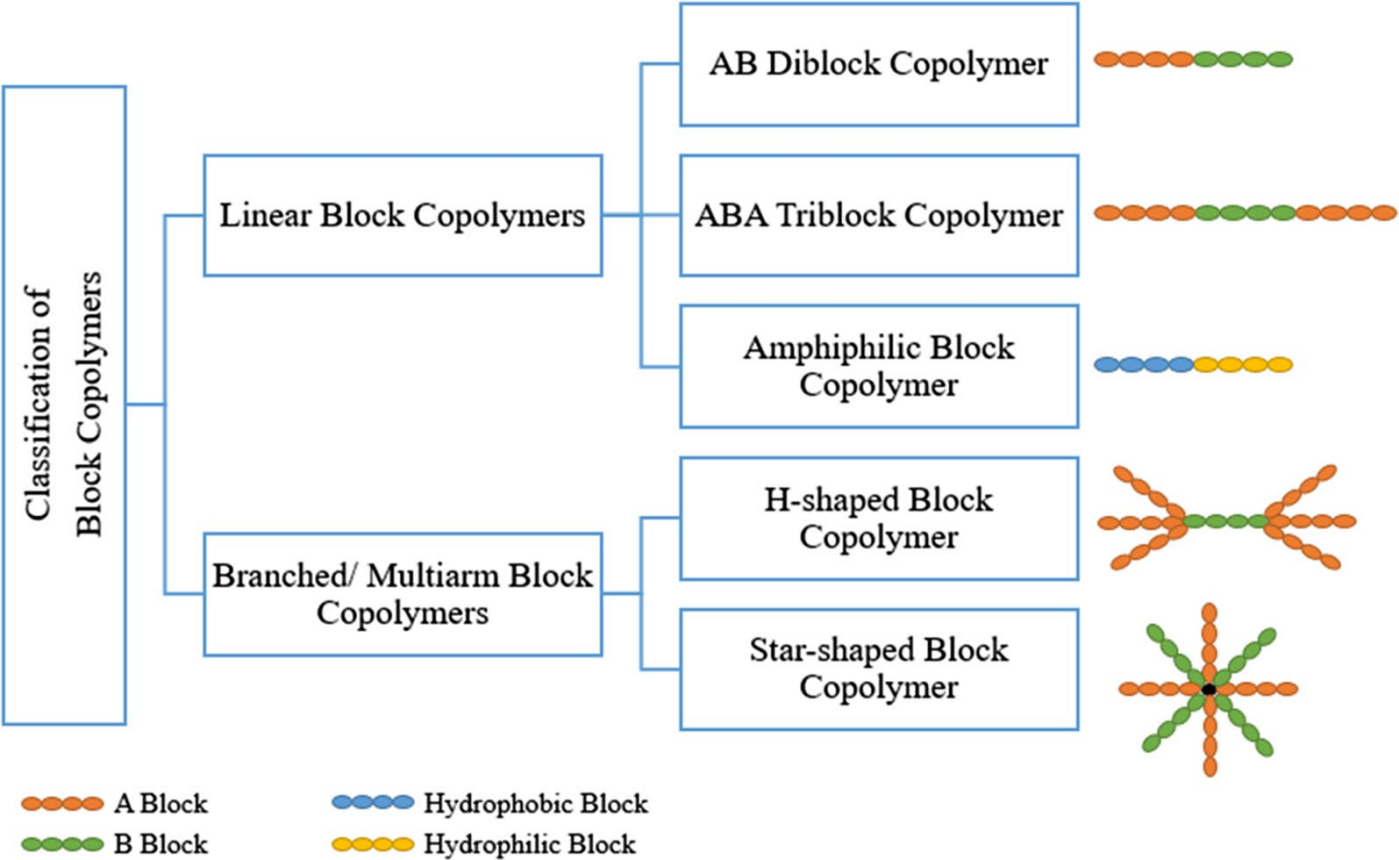
Types of block copolymers

Based on the arrangement of the blocks, the block copolymers can be of different types. They are di-, tri-, multi-, and multi-arm block copolymers (Fig. 2). Diblocks consist of two different blocks (A, B) arranged in ‘AB’ manner while triblocks are arranged in ‘ABA’, ‘BAB’ or ‘ABC’ manner. A, B blocks can be further arranged to form alternating and tapered block copolymers [12]. Amphiphilic block copolymers are composed of hydrophobic and hydrophilic parts which cause them to cluster and form micelles and can be used in drug delivery and tissue engineering [13]. Multi-arm block copolymers have several branches attached to a common core and some of them are H-shaped [14], star-shaped [15], miktoarms [16].

It has been found through experimental and theoretical studies that block copolymers can separate into different forms like spheres, cylinders, lamellae and bicontinuous structures. Factors affecting these forms include copolymer composition, w-parameters and the total degree of polymerization [12].

### Synthesis

**Fig. 3:**
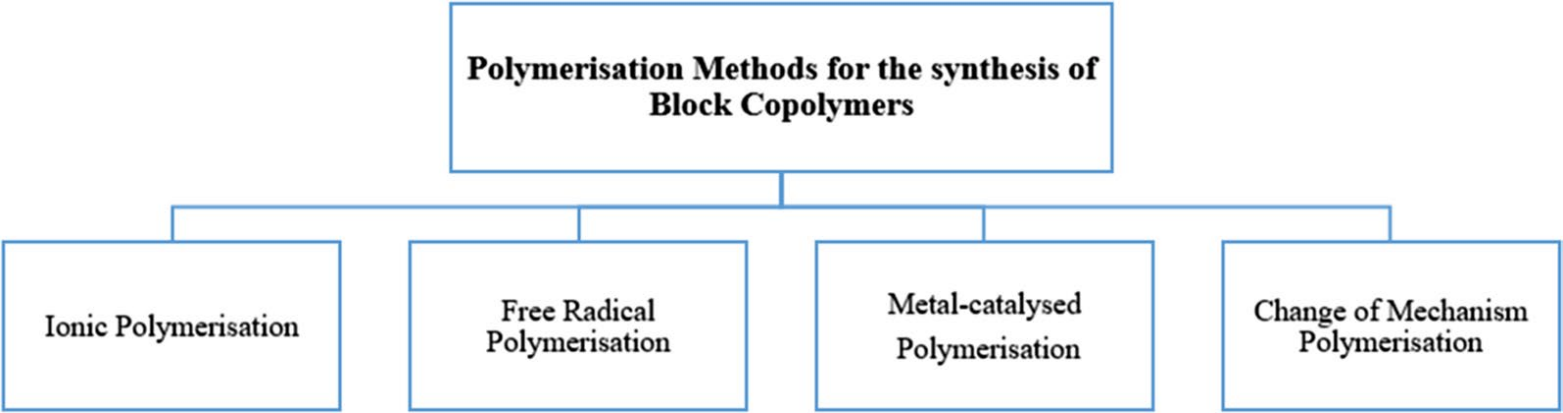
Polymerisation methods for the synthesis of block copolymers

Block copolymers especially diblock and triblock copolymers are generally synthesised through living or controlled polymerization of the monomer. The techniques used are ionic polymerisation, free radical polymerisation, metal catalysed polymerization and change of mechanism polymerisation (CHOMP) (Fig. 3). Anionic and cationic polymerizations are chain polymerizations that use an ionic group as active polymerization centre. These groups are highly reactive and special precautions are necessary to prevent any side reactions. Styrene, diene (monomers) block copolymers with polyacrylate groups are prepared by anionic polymerization. Cationic polymerization is suitable for preparing block copolymer monomers like styrene, vinyl ethers and isobutylene [17]. Free radical polymerization causes living polymerization through a free radical group. With this technique, block copolymers can be prepared with monomers that are deemed to be unsuitable for metal catalysed or ionic polymerizations. TEMPO (2,2,6,6-tetramethylpiperidinyl-1-oxy) mediated and RAFT (reversible addition–fragmentation chain transfer) living free radical polymerisation are some of the techniques used for the synthesis of block copolymers [17–19]. Metal catalysed polymerization uses a metal catalyst for polymerization. This technique is adaptable to many monomers and can be carried out under different reaction conditions. Kharasch addition reaction or atom transfer radical addition and metal catalysed living free radical polymerisation are some of the techniques in this type of polymerization [20]. CHOMP is a technique which can be accomplished through in situ conversion of the active centre or by separating the first segment followed by transformation of the active centre during the reaction. Sequential addition of monomers is necessary in both the cases because the active centre of the first monomer can be deactivated or destroyed by the second monomer [17].

### Properties

Block copolymers as compared to other copolymers have superior properties and these properties highly depend on the clustering and structural arrangement of the blocks. They allow the combination of significantly high melting points and elastic properties in the same polymer. Block copolymers also exhibit increased tensile strength, modulus, and elongation by suitable selection of the blocks. Other properties like moisture recapture, ability to absorb dyes also depend on the block copolymer’s structure [21]. The phase behaviour of a block copolymer is dependent on the following factors: overall degree of polymerisation (N), architectural constraints (n) and composition (ƒ) and the Flory–Huggins interaction parameter (χ). The first two factors influence translational and configurational entropy of the copolymer. Increasing χ by lowering the temperature results in less A-B monomer contact. Large N causes some loss of configurational and translational entropy and causes microphase segregation. Decreasing χ and N causes the formation of compositionally disordered phase [22]. In a study, a series of symmetric EPE triblock copolymers were investigated to study the effect of microphases separation on microstructure and mechanical properties of solid state chain. Block copolymers having crystallizable components can undergo microphase separation either due to chemical incompatibility between the blocks causing the formation of a heterogeneous melt or through the expulsion of a block due to crystallisation of another block. The latter is also known as crystallisation induced microphase separation. The tensile strengths of these triblock copolymers depend on the manner of microphase separation. Crystallization-induced segregation causes lower tensile strength than those with templated crystallisation [23]. To realise the actual potential of block copolymer nanostructures, several strategies are required to control and manipulate spatial orientation, connectivity, periodicity, and long range order [11, 24–27]. Topologically [28] and chemically patterned surfaces [29], solvent annealing [30, 31], temperature gradients [32] and electric fields are some of the techniques which utilize physical and chemical constraints to direct self-assembly of block copolymers into highly well-ordered geometries. Electric fields are a versatile and easy way to control long range orientational and translational order of nanostructured block copolymers as well as miscibility and morphology both in thin films, bulk and solutions [33].

## Nanostructures and block copolymers

Block copolymers create well-ordered structures by undergoing microphase separation. Their properties can also be controlled through manipulating the three critical parameters mentioned above. Block copolymers can be used to synthesise different nanostructures due to their fitting size and shape. Their properties are also easily tunable by simply changing the monomer used, their compositions and molecular weight. Using block copolymers for fabricating nanostructures is simple and affordable as well [34]. This section briefly focuses on synthesis of nanowires, nanorods, nanoribbons and nanofibers using block copolymers.

### Nanowires

**Fig. 4:**
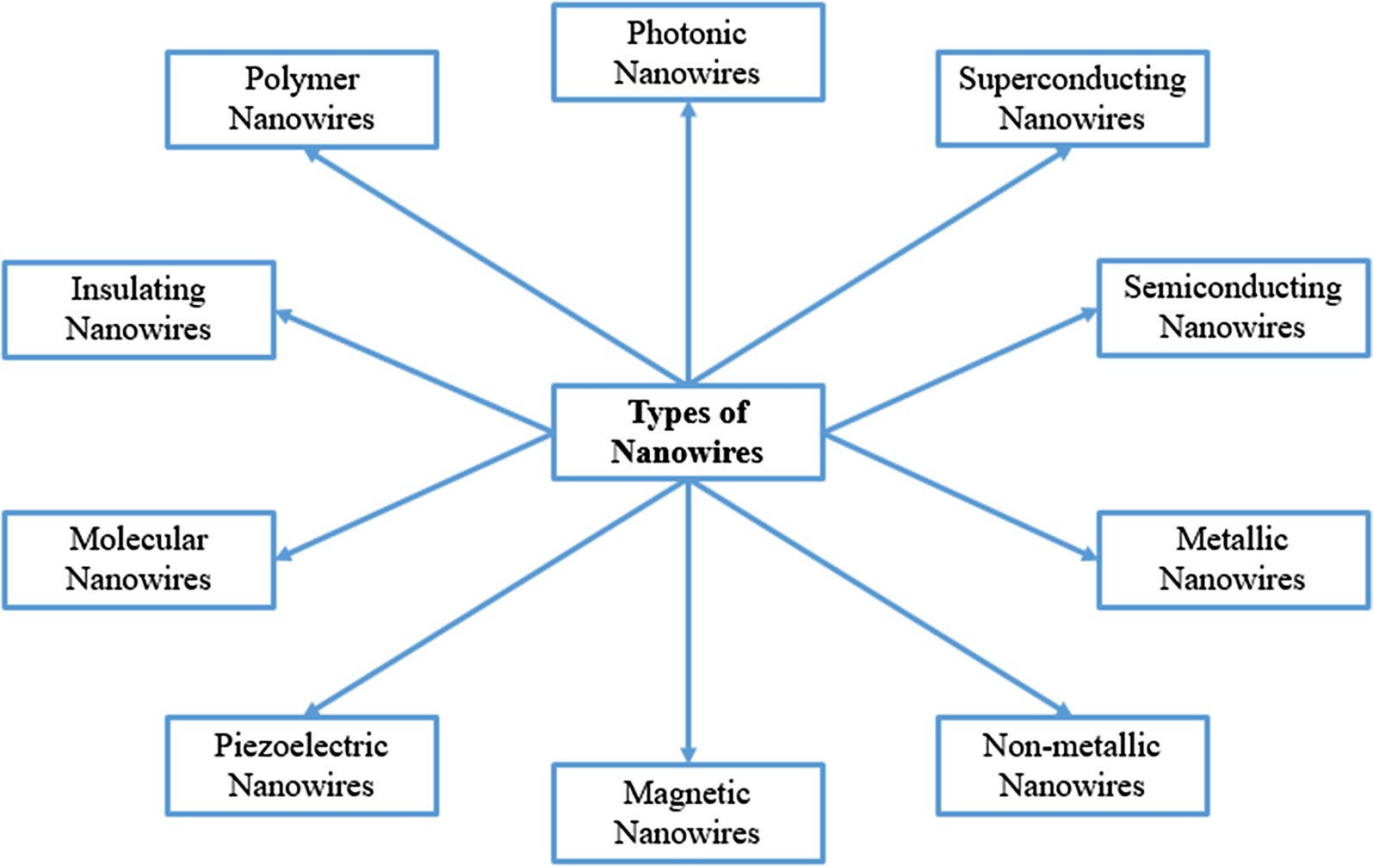
Types of nanowires

Nanowires are one-dimensional nanostructure with a very high aspect ratio and a diameter in the nanometer range. Such a dimension allows nanowires to manipulate and control various subatomic particles like electrons, photons, phonons etc. to give rise to different applications [35–38]. Based on their properties and the source used to fabricate nanowires they can be of many types. They are semiconducting, photonic, superconducting, metallic, non-metallic, insulating, polymer, magnetic, piezoelectric, and molecular (Fig. 4).

**Fig. 5:**
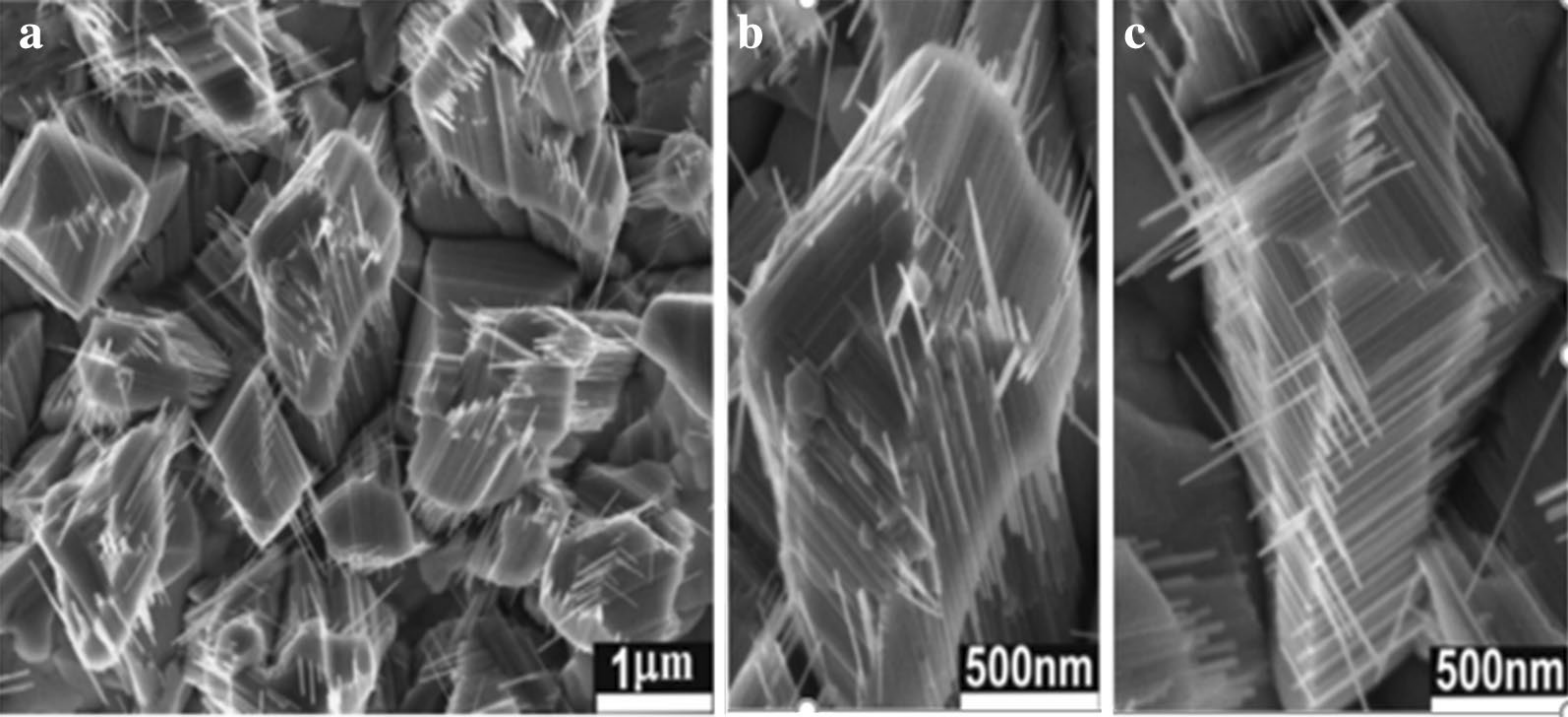
**a** Low magnification SEM images of Mn_3_O_4_ nanostructures/nanowires. **b**, **c** High magnification SEM images of Mn_3_O_4_ nanostructures/nanowires [41]

Each type of nanowires have unique properties that are useful in many applications. Literature has provided many techniques for the synthesis of nanowires. Methods vary slightly for different nanowires. Synthesis occurs generally through electrochemical methods, synthetic chemical routes, nanoimprint lithography, template assisted techniques, and chemical vapor deposition technique [39]. Metal nanowires like cobalt have been synthesized using block copolymers. Diblock copolymers such as polymethylmethacrylate (PMMA) or polystyrene were used as templates. These polymers were subjected to various conditions and made into films through spin coating techniques followed by vacuum annealing. Application of electric current for a longer duration, oriented the block copolymer in the direction of the field followed by exposure to UV radiation to cut down template followed by further treatment with acetic acid. Such a treatment caused the formation of cylinders on the template of the nanoscale region which were further characterized using SEM. These templates were also used for the synthesis of Cobalt nanowires through electrodeposition [40]. Metal oxides like manganese oxide (Mn_3_O_4_) have been fabricated into 3-D crossing nanowires using soft chemistry templating synthesis. Here, Manganese chloride was filtered and mixed with block copolymers to form a sol of the same. The resultant sol was then aged overnight and a change in color was observed. This sol was then deposited on the silicon wafer which was then followed by calcination. These samples, were then characterized by SEM (Fig. 5), TEM, and XRD. The magnetic properties of these wires were also measured through Super Conducting Quantum Interference Device (SQUID) magnetometer. Characterization studies revealed unique nanostructures in which nanowires remained spatially orthogonal in 3-D. They also showed some nanostructures that had nanowires forming a spatially perpendicular 2-D nanowire network. It was also revealed that the structures were like the spinel structure of Mn_3_O_4_ [41].

Metal oxalate nanowires like copper oxalate have also been synthesized using block copolymer. Block copolymer material in samples of SBA-15 was used as a reactant. This was oxidized to C_2_O_4_^2−^ in a special aqueous solution containing copper ions. Due to this, copper oxalate nanowires embedded in mesoporous silica channels were produced in situ. Through heating of CuC_2_O_4_/SBA-15, CuO and Cu_2_O nanowires were also produced. These nanowires demonstrated ordered mesostructures and good textural properties through characterization studies using SEM, TEM, and FT-IR. They also possessed excellent electrochemical hydrogen storage capacity [42]. Nanoporous templates have been created using diblock copolymers consisting of different ratios of block copolymers. Gold coated silicon wafers were then spin coated on this template followed by vacuum annealing and intense exposure to UV radiation. In this nanoporous template, NiFe alloys were electrochemically deposited. NiFe has magnetic properties and can be used in magnetic storage devices and as sensors for gas. The wires were further characterized by SEM [43]. Through this technique, molybdenum disulfide nanowires have been fabricated as well. In a study, self-assembled cylindrical diblock copolymer thin films were used to synthesize these wires. It employed selective seeding of ammonium heptamolybdate (AHM) or ammonium tetrathiomolybdate (ATTM) precursors into specific domains of the diblock copolymer [poly(styrene-*b*-2-vinylpyridine)] thin film. This was later annealed to synthesize molybdenum disulfide nanowires on SiO_2_/Si substrates. The experimental conditions were modified in such a way that they produced either polycrystalline or amorphous molybdenum disulfide nanowires. These were later characterized through TEM, Raman spectroscopy and X-Ray Photoelectron Spectroscopy (XPS) and XRD. It revealed that at higher annealing temperatures there was a transition from amorphous phase to polycrystalline phase with a decrease in excess elemental sulfur [44].

**Fig. 6:**
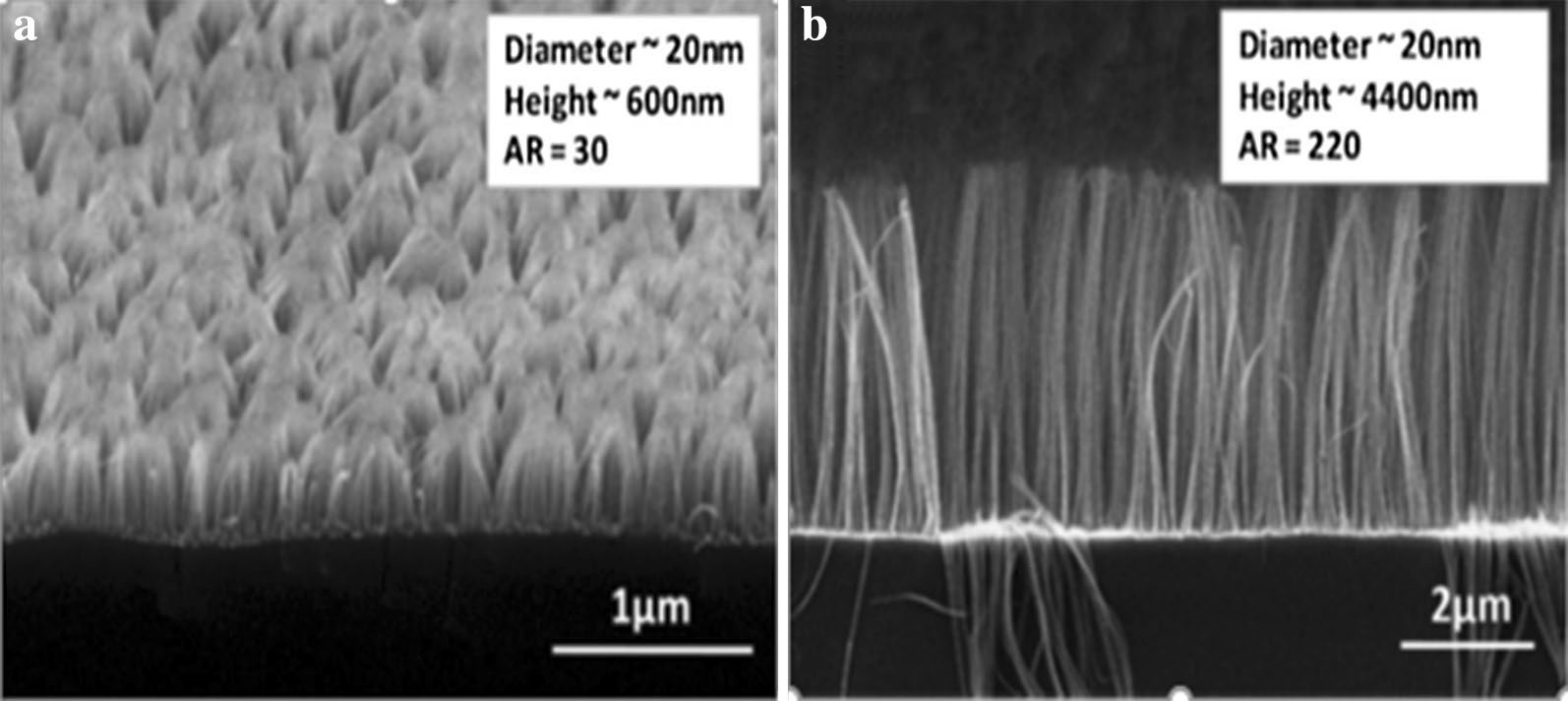
SEM images of silicon nanowires. **a** With AR of 30, **b** with AR of 200 [53]

Block copolymer lithography is based on the self-assembly of block copolymer molecules into periodic microdomains, on the 10–100 nm scale. Here, block copolymers can form structures that are lithographically transferred into other materials such as semiconductors and metals to form complete devices. It is an excellent strategy for economically evaluating dense and large area arrays of nanoscale structures [45]. It also generates nanowires of uniform size and can use conventional microscale lithographic techniques which can spatially control nanowire growth through careful patterning of catalyst coverage [46]. Block copolymers have been used to generate one dimensional nano-assembled structures along chemically striped patterned surfaces. The lateral dimension of the nano-assembled structure was in the 20 nm scale, while the dimension of the chemically striped pattern directing the assembly was in the range of 70–150 nm. This approach is excellent to hierarchically control the self-assembled nanoscale morphology over the usual lithographic processes [47]. Aligned block copolymer self-assembled structures have been used for the synthesis of linear metallic nanostructures of platinum, palladium, and gold on silicon. Here, metallic lines of nanostructures were produced by taking advantage of the acid-responsive nature of block copolymers and loading metals in it through aqueous solutions of anionic metal complexes. The acid-responsive nature of block copolymers causes them to swell on exposure to acid, which leads to a direct contact with the aqueous metal solution. These anionic metal salts then electrostatically bind to the block copolymer. Metallic nanostructures are formed after reduction of the hybrid structure and the removal of polymer through plasma treatment. Parallelly arranged metallic lines and concentric rings were obtained with sub 50 nm spacing [48]. Another approach to synthesize metal nanowires involved creation of one-dimensional arrays of block copolymers with careful control of the block copolymer film thickness within a topographic confinement of photoresist pattern. These photoresist patterns were prepared by I-line lithography and the block copolymer assembly generated 20 nm thick lamellar stack. Advantages of these techniques include large scale production of densely packed nanowire array [49]. Another nanolithography technique is soft graphoepitaxy in which organic negative-tone photoresist was used to direct block copolymer assembly. In this technique, the photoresist was uniformly spin-coated on a substrate and topographically patterned by conventional lithography. A thin film of diblock copolymer (PS-*b*-PMMA) was spin-coated on the topographically patterned surfaces and annealed at high temperatures. Post annealing, the PMMA domains were selectively dry-etched from the block copolymer thin film. Following pattern transfer, the substrate surface now had highly ordered nanopatterned surfaces with desired shape and orientation [50]. Ultrathin silicon nanowires have been prepared using block copolymers [poly(styrene)-*b*-poly(methyl methacrylate)] using this technique for electrooxidation and sensing of ethanol. This was done by using the low molecular weight block copolymer to create a nanopattern which was transferred to the substrate through plasma etching. The orientation of the block copolymer was controlled through polymer brushes which were hydroxyl terminated. The silicon nanowires produced were characterized by SEM, TEM, and Energy Filtered Transmission Electron Microscopy (EFTEM). These studies revealed nanowires in sub-nm range with a high degree of domain alignment [51]. Block copolymer lithography has also been used to fabricate conducting polymer nanowires. They were prepared on a well-ordered array by using an etch mask consisting of self-assembled patterns of cylinder confined in a topographic template. These wires were then operated as an ethanol vapor sensor, which showed that the electronic properties of the organic mask were preserved during the patterning process [52]. Silicon nanowires with high aspect ratios have ben fabricated through metal-assisted etching in conjunction with block copolymer lithography (Fig. 6). This technique can also be applied to other physical template geometries. This technique also exhibited extensive flexibility in the placement of wires at desired location over a large area. It also has excellent control on the size, placement accuracy, and geometry of the block copolymer microdomains [53].

**Fig. 7:**
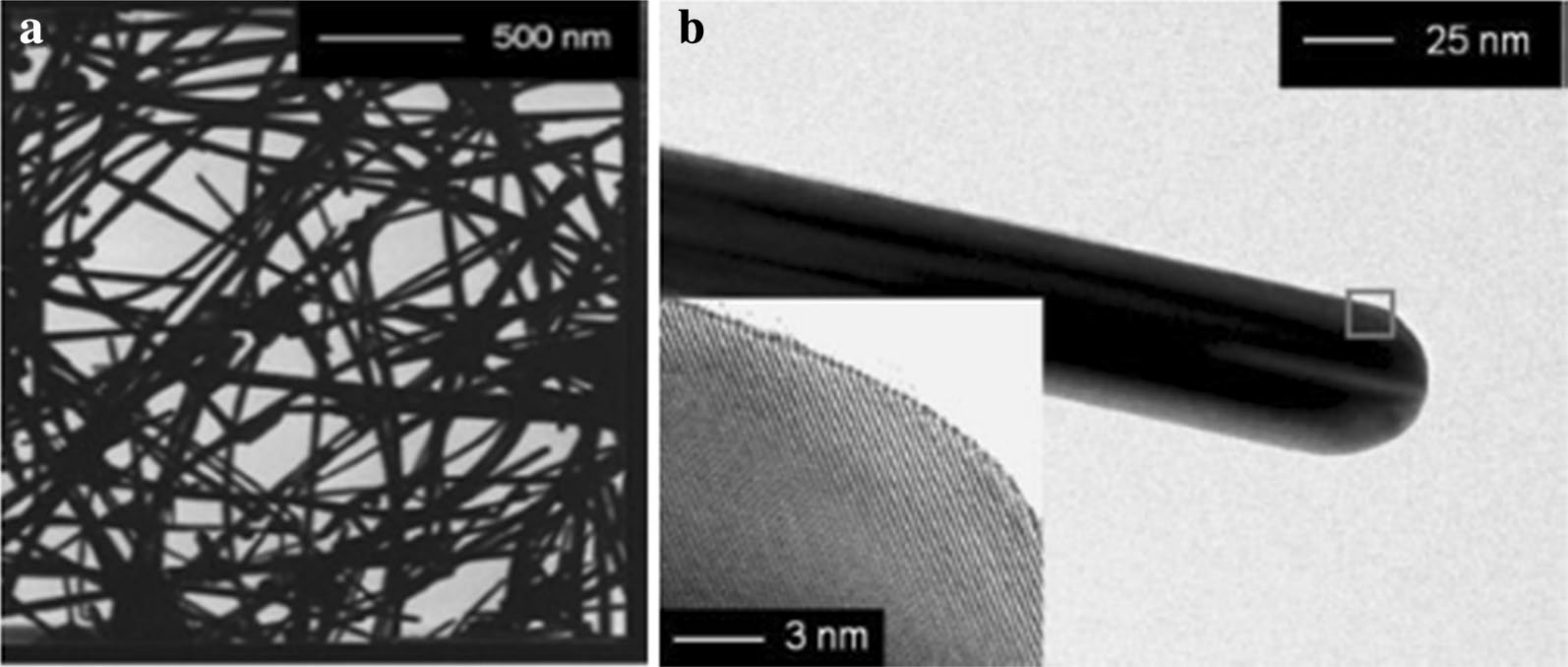
**a** TEM images of gold nanowires, **b** *inset* shows high resolution image of gold nanowires [55]

Block copolymers can be also used in solution form for the synthesis of nanowires. Double-hydrophilic block copolymers have been used for the synthesis of silver nanowires. They have hydrophilic blocks which interact strongly with appropriate inorganic materials and promote solubilization in water. Here, AgNO_3_ solution was added to the diblock copolymer solution and allowed to stand for 54 h. Development of a yellowish color indicated the formation of silver nanowires. The formation of these nanowires was spontaneous and didn’t require any further treatment with ultraviolet irradiation or any electrochemical method. These wires were then characterized by UV–visible spectroscopy and TEM. The images obtained after TEM analysis revealed many silver nanowires with diameters in the nanometer range. They also exhibited distorted or curved morphology [54]. Gold nanowires can be synthesized through block copolymers as well. In one study, the synthesis took place through two basic processes: Preparation of mixture and reduction. The block copolymer used served as a reaction medium as well as a capping agent for the formation of nanowires. Photoreduction through UV rays and the thermal reduction was used to reduce the gold salt in the block copolymer solution. THF and water was also added to slowly homogenize the solution. The solid mixture left behind post the removal of THF was further exposed to UV radiation to reduce the gold salt to gold nanostructures. These nanostructures, when characterized by TEM, revealed gold nanowires along with other 2D structures. SEM and Small-Angle X-Ray Scattering (SAXS) were also used for characterization of these nanostructures (Fig. 7) [55].

**Fig. 8:**
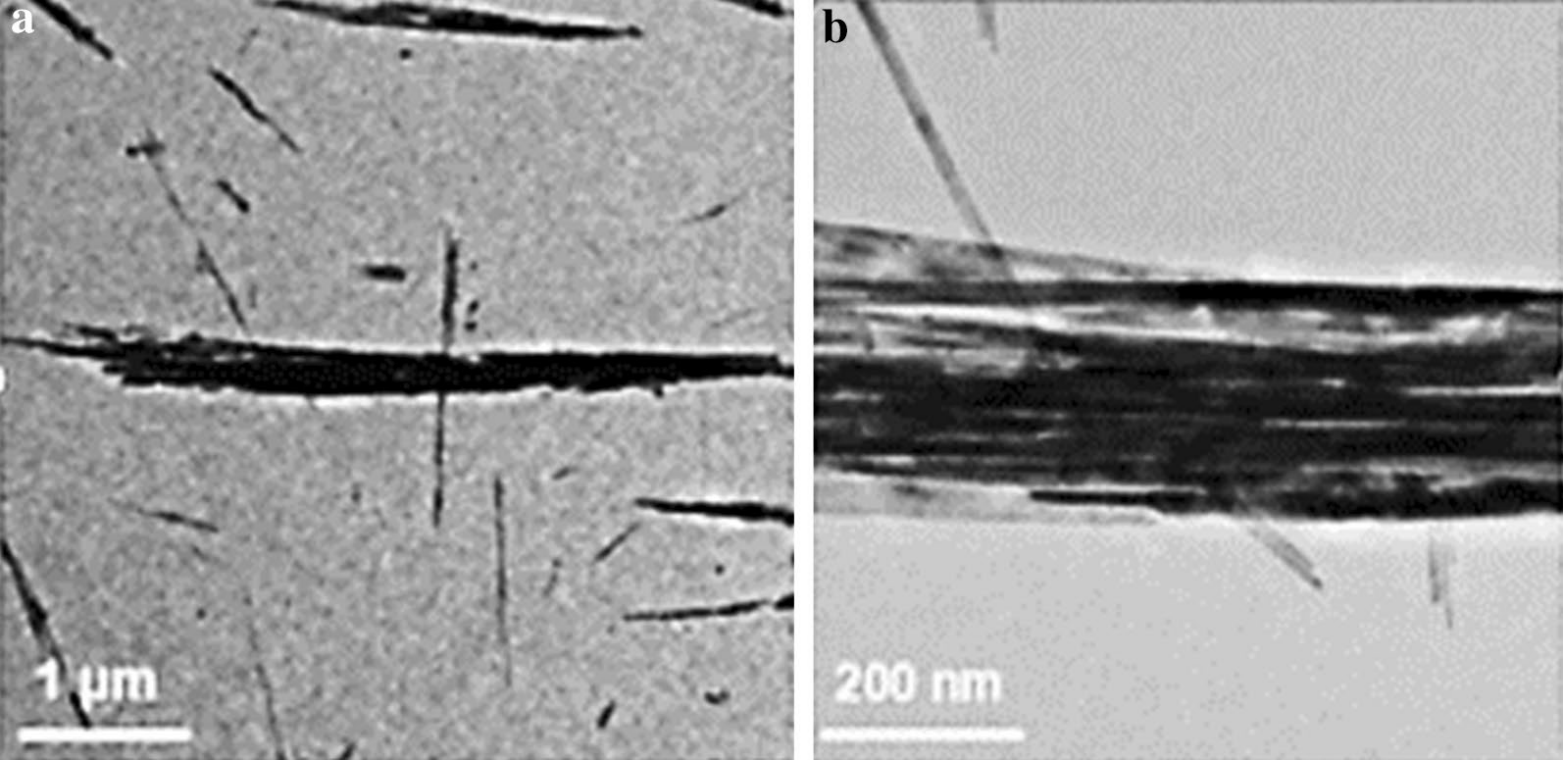
**a**, **b** TEM images of Cu_2_O nanowires [56]

Metal oxide nanowires like copper oxide have been synthesized using triblock copolymer solution media. In this study, a triblock copolymers solution was added to a mixture of CuCl_2_ followed by stirring for a certain period. Reducing agent was added to the mixture and the entire system was heated to 25 °C. Cu_2_O nanowires were obtained through rotary evaporation procedure. XRD, TEM, Differential Scanning Calorimetry (DSC), and Thermogravimetric Analysis (TGA) was used to characterize these nanowires. They revealed copper oxide nanowire in 8–10 nm range, but of non-uniform diameter. Factors affecting the nanowires were the concentration of triblock copolymer and aging period of the solution. Cu_2_O nanowires with diameter mainly in the 8–10 nm range were obtained (Fig. 8) [56].

### Nanorods

Nanorods are nanostructures with characteristic size dependent properties whose effects are significantly observed in the 1–10 nm range. Nanorods also can cause large variations in its composition. They exhibit typical optical, electronic, and catalytic properties which cause them to have many applications. Nanorods like any other nanostructures are also synthesized through ‘top-down’ and ‘bottom-up’ approach. Techniques generally used to synthesize these nanorods include seed-capping, vapor–liquid–solid growth, chemical vapor deposition, solid controlled growth, electrodeposition, sol–gel process, lithography, self-assembly, and template-based synthesis [57].

**Fig. 9:**
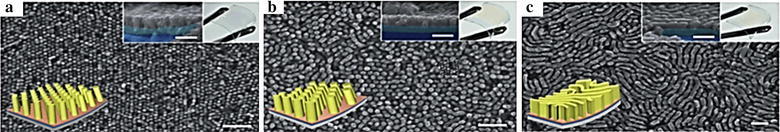
**a**–**c** FE-SEM images of ZnO nanorods grown from different templates. The *yellow* schematic images show the pore size of each template. *Inset* images show the* side view* and the flexible substrate held by tweezers [58]

Template-based synthesis has been used for the synthesis of block copolymers using nanorods. ZnO nanorods have been fabricated by combining hydrothermal growth technique with nanoporous block copolymer templates. Templates with cylindrical nanopores in two sizes were fabricated with an indium tin oxide (ITO) electrode and covered with an amorphous ZnO seed layer. From this template, vertically oriented and uniform ZnO nanorods were synthesized by exposing the template to oxygen plasma which removed the adhesive layer of ZnO seed in the pores and made the template hydrophilic. Hydrothermal growth was later carried out to obtain the nanorods. FE-SEM images revealed the average diameter of the pore size to be 13 nm (Fig. 9). It also revealed nanorods with a diameter larger than the pores due to the probable softening of the template during the hydrothermal growth of ZnO nanorods. Since these nanorods were directly produced on ITO electrodes, the semiconducting nature of these nanorods was also characterized which revealed the typical rectifying behavior of the nanorods [58].

Nickel nanorods have been prepared by applying a mask of ordered nanostructured hollow channels in a block copolymer matrix. Through an organized process in block copolymer supramolecular assemblies, the polymeric templates were formed. Onto this template, nickel was deposited via two techniques: electrodeposition and washing-in; and the entire formation process of the nickel nanorods was monitored through AFM and X-Ray Photoelectron Emission Microscopy (XPEM). Through this, it was found that the nickel rods showed metallic behavior despite being synthesized under ambient conditions. Also, no NiO complexes were formed due to the probable protection of Ni nanoparticles against oxidation [59].

**Fig. 10:**
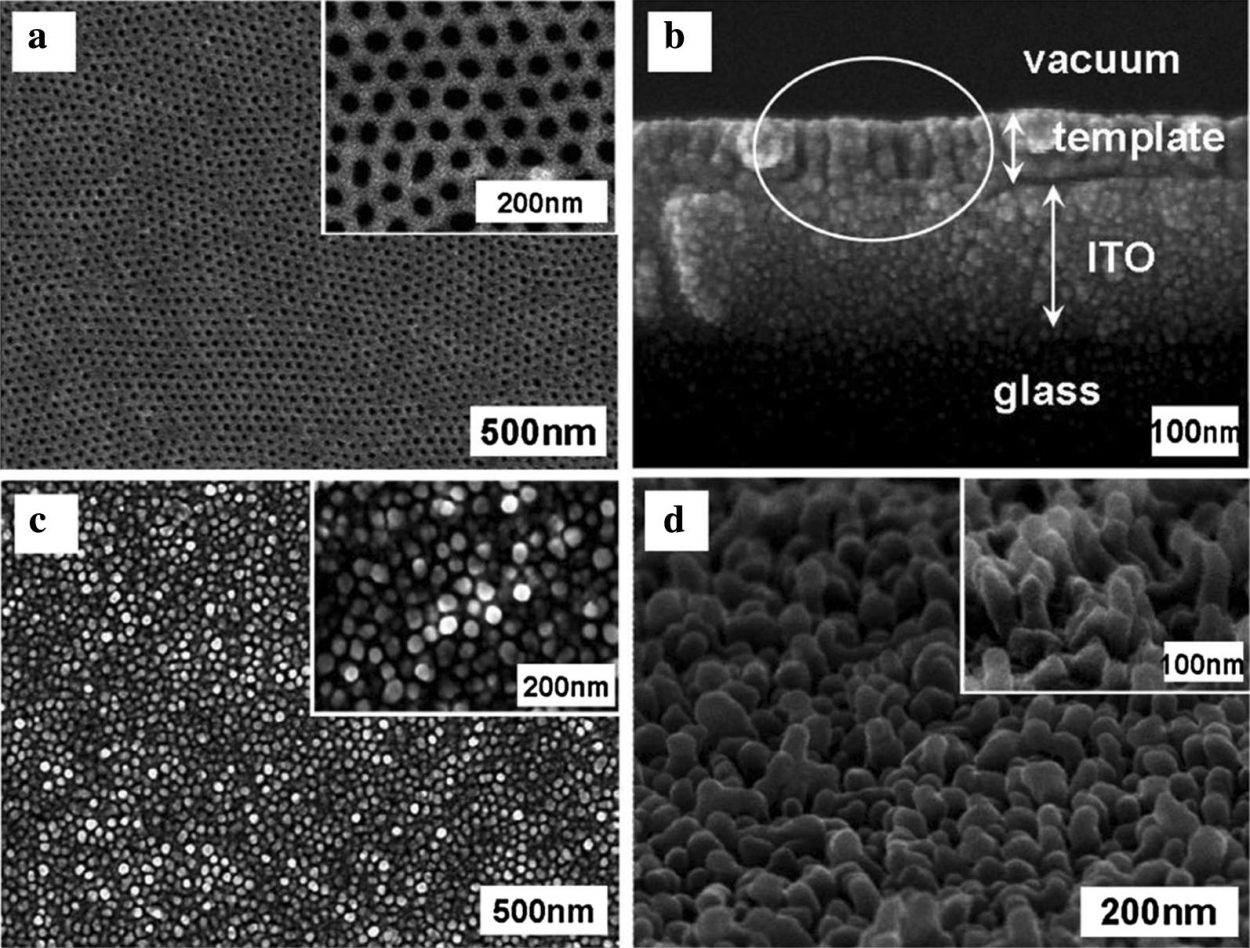
FE-SEM images of nanoporous templates prepared by the block copolymer mixture film on the ITO glass and polypyrrole nanorods. **a**, **b** Top and cross-sectional images of the nanoporous template. **c**, **d** Top and cross-sectional images of polypyrrole nanorods after removing the template [60]

Ultrahigh density arrays of nanorods of polypyrrole (PPy) have been synthesized directly on ITO coated glass through electropolymerization within a porous diblock copolymer template. Here, ITO-coated glass was first spin coated with a hydroxyl-terminated random copolymer. Post washing of the random copolymer, PS-*b*-PMMA block copolymer was spin coated on the glass. Through UV radiation and treatment through acetic acid, the PMMA part of the block copolymer was removed which formed the nanoholes for growing the conducting polypyrrole nanorods. The PS-*b*-PMMA matrix was removed by toluene, leaving behind highly aligned ultrahigh density of self-supporting conducting polypyrrole nanorods. Characterization studies by FE-SEM revealed cylindrical pores that were oriented normally to the ITO glass. These pores had diameter lesser than 25 nm which enabled the formation of nanorods. These nanorods also had conductivity significantly higher than thin PPy films due to the high degree of chain orientation (Fig. 10). Potential uses of these nanorods can be found as sensor materials organic photovoltaic devices and nanoactuators [60].

**Fig. 11:**
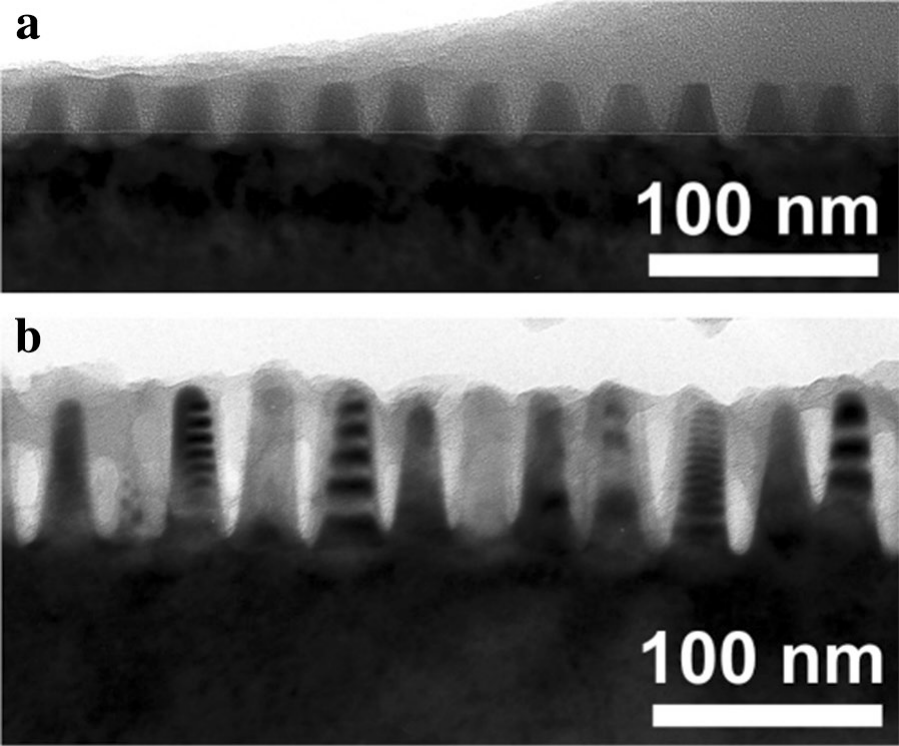
TEM cross-sectional image of Si nanorods. **a** Underlying Si wafer connected with Si_3_N_4_ nanodots, **b** Si nanorods connected to underlying Si wafer after etching of the exposed Si [61]

Block copolymer lithography has been used to fabricate nanorods as well. In a study, ordered arrays of Si nanorods with uniform aspect ratios of 5:1 and a diameter of 15 nm were fabricated through block copolymer lithography. Arrays of cylindrical polystyrene (PS) domains instead of the generally used porous continuous films were fabricated. A layer of Si_3_N_4_ was deposited on the silicon wafer through CVD. Hydroxyl-terminated random copolymers were grafted on the layer of Si_3_N_4_ followed by annealing for 40 h at 165 °C in the presence of nitrogen. The PMMA matrix was removed by exposure to UV radiation. To enhance the contrast in following dry etching steps, the PS domains were exposed to RuO_4_ vapor. The exposed Si was later etched to form Si nanorods (Fig. 11) [61].

ZnO nanorods have also been synthesized in a solution based approach. Effect of the diblock copolymer poly(ethylene oxide)-*b*-poly(propylene oxide) (PEO-*b*-PPO) has been investigated on the morphological control of radial spherical ZnO nanorods. Solutions of different molar ratios of the block copolymer was added to the aqueous solution of Zn(C_2_H_3_O_2_)_2_·2H_2_O followed by addition of NaOH solution to the PEO-*b*-PPO modified zinc solution. SEM was used to further characterize the precipitate obtained to reveal ZnO nanorods. The length and the diameter of the hexagonal facet of each rod decreased with increase in copolymer concentrations. The effectiveness of photocatalytic degradation of the nanorods also increased with increase in their surface areas. However, there was no improvement in the antibacterial activity of the nanorods due to their sizes [62]. In another solution based approach, size-tunable CdS nanorods were synthesized at low temperature via the reaction of air-insensitive inorganic precursors sodium sulfide and cadmium acetate in an aqueous phase in the presence and absence of a surfactant. Here, nonionic amphiphilic triblock copolymers were used as structure-directing agents. In the absence of surfactant, CdS nanorods were synthesized by refluxing at high temperatures using ethylene glycol, ethylene glycol dimethyl ether or a mixture of the two chemicals as the solvent. The inorganic precursors were added followed by refluxing which led to the formation of a yellow precipitate. In the presence of a surfactant, the amphiphilic triblock copolymer was dissolved in deionized water at different ratios. The precursors were added in the solvent and the entire reaction was carried out in a N_2_ atmosphere. The temperature was gradually increased and the resulting solution was centrifuged to obtain yellow to reddish orange precipitates. These were then washed with ether/ethanol mixture. SEM analysis revealed a uniform flat-ended rod whose average length was greater than 4 µm and diameter of 0.2–0.6 µm. It was also found that the diameter of the CdS nanorods were easily controlled by changing the surfactant species. However, in the absence of a surfactant, the morphology of the product changed to microrods with flat ends, cotton-ball microparticles, and dumbbell-shaped microrods. The typical diameter of the nanorods obtained were 5–7 nm and was 30–90 nm in length [63].

### Nanoribbons

Nanoribbons are 1-D nanostructures that have gained attention due to their unique flat geometries which enable them to prompt positive changes in terms of pore size distribution and shape. It also enables them to improve their anisotropic mass and heat transport and strengthens their mechanical properties [64]. This section briefly focuses on the synthesis of graphene nanoribbons through block copolymers.

Graphene nanoribbons are strips of graphene with dimensions less than 10 nm [65]. Lithographic, sonochemical, chemical and unzipping methods like high current pulse burning and catalytic cutting methods have been developed to fabricate graphene nanoribbons [66].

**Fig. 12:**
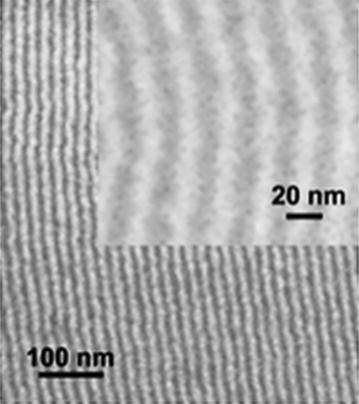
SEM image of GNR array. *Inset* GNR under high magnification with nanoribbon width around 8 nm [67]

Fabrication of sub-10 nm line patterns from a lamellar diblock copolymer polystyrene-*b*-polydimethylsiloxane (SD) has been done through block copolymer lithography. Thin SD films directly spin cast onto silicon and graphene substrates create regular line patterns of sub-10 nm pitch after annealing at 45 °C in the presence of toluene vapor. This template was further used as a lithographic mask to fabricate sub-10 nm graphene ribbons of high quality. Etching of the PS block, hardening of PDMS block by oxidation and etching of graphene under the PS domains were realized through application of oxygen plasma on the spin cast and the annealed block copolymer. Characterization was done with SEM and Raman analysis which revealed the formation of graphene nanoribbons with no defects in the ribbon channel due to the high degree of protection of the nanoribbons by the hardened oxidized PDMS mask formed post application of plasma oxygen (Fig. 12) [67].

In another study, FET characteristics and photoelectric properties of graphene nanoribbons having 9–12 nm ribbon widths that were fabricated through cylindrical PS-*b*-PDMS block copolymer as a lithographic mask to pattern graphene single layers of graphene grown through CVD on Si substrates which were heavily p-doped with an SiO_2_ layer was demonstrated. Here, a PEO brush replaced PDMS brush to prevent the formation of a PDMS layer at the graphene surface. The graphene nanoribbons fabricated into FETs through this technique displayed a higher on/off ratio and a stronger temperature dependence of the current as compared to the FETs having no patterned graphene nanoribbons [68]. Graphene is atomically thin which causes the development of a unique surface energy characteristic called ‘wetting-transparency (WT)’. This property can be exploited for graphene nanoribbon array. A research group has used this property to demonstrate a robust procedure to fabricate graphene nanoribbons by placing the cross-linked SNT (Surface Neutralized Treatment) layer under the graphene, where the SNT can control the orientation of block copolymer due to WT. The procedure involved the growth of graphene monolayer on a copper foil followed by transferring of this monolayer onto an SNT-coated Si wafer through PMMA-assisted transfer method. The diblock copolymer PS-*b*-PMMA [poly(styrene)-*b*-poly(methyl methacrylate)] was spin-coated on the mild plasma treated graphene substrate followed by annealing to achieve perpendicular domain orientation. The PMMA domain was then selectively removed through a CO_2_ based etching process and the pattern formed was subsequently transferred into the underlying graphene. The residual PS was removed by soaking it in THF, followed by drying of the sample at room temperature under vacuum. Characterization studies by AFM and SEM revealed successful removal of the PMMA block and SNT layers. Since graphene is placed between the block copolymer and SNT, this observation showed the successful etching of graphene thus transforming it into graphene nanoribbons. Also, the structure of graphene nanoribbons was identical to the PS fingerprinting pattern, thus confirming the pattern transfer from the block copolymer mask to fabricate the nanoribbons [69].

### Nanofibers

**Fig. 13:**
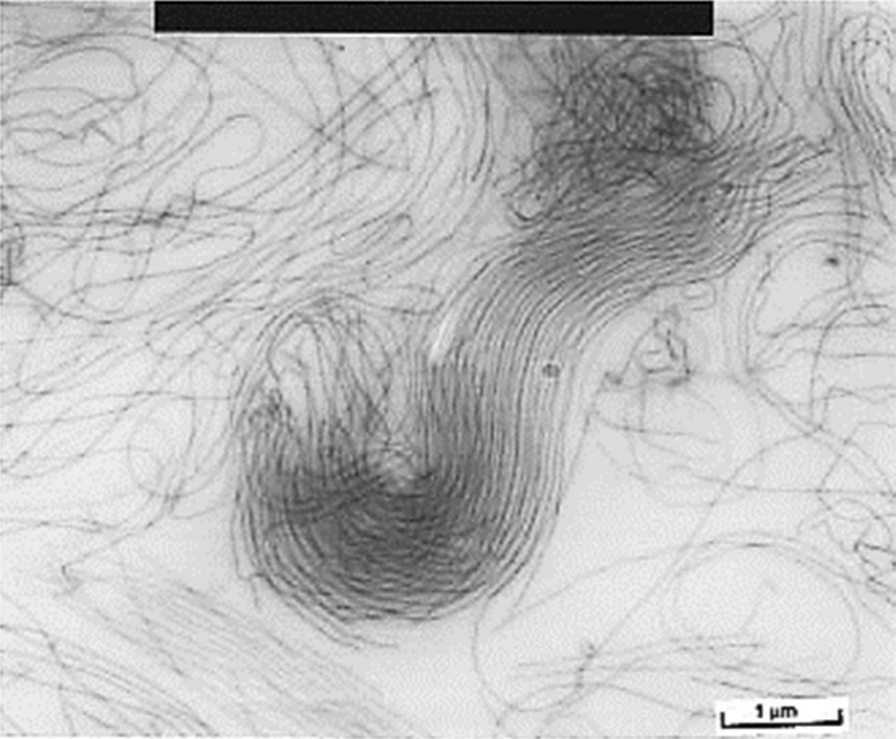
TEM image of diblock nanofibers [71]

Nanofibers are defined as fibers with diameters less than 100 nm. They can be made from a variety of materials like carbon, semiconductors, polymers, and cellulose and find applications in tissue engineering, reinforcement in composites, filter media and micro/nano-electro-mechanical systems. Conventional synthesis techniques for nanofibers involve template based, electrospinning, nanoimprint lithography, and electrochemical approaches [70]. Diblock copolymer nanofiber is defined as an aggregate of a diblock copolymer which is cylindrically shaped. The structure of the nanofiber in a solvent-free state comprises of a crosslinked core and concentric shell made of blocks. On dissolving in a good solvent, the chains of the block stretch out and swell. The strategy used to fabricate diblock copolymers involved preparing bulk samples from a diblock copolymer with a cross-linkable block as the cylindrical phase. TEM images revealed cylindrical phase formation from the PCEMA [poly(2-cinnamoylethyl methacrylate)] block. Post establishment of cylindrical phases of the block copolymer, the nanofibers were prepared by annealing the polymer disks and irradiating it with UV. Later, a thin film produced was transferred to the copper grid of TEM for viewing. It revealed nanofibers that were 25 nm thick and thousands of nanometers long (Fig. 13). Nanofiber bundles were observed as well [71].

Self-assembled block copolymer nanofibers are excellent materials having applications as viscosifying agents in complex fluids or in biomedical field. The preparation of polymeric nanofibers is based on an aqueous emulsion polymerization using the RAFT method. RAFT agents were synthesized followed by the synthesis of the block copolymers using these RAFT agents under different pH and salt concentrations. The effect of these parameters on the formation of nanofibers was studied by TEM. With RAFT agents, at acidic pH or high salt concentrations, non-spherical morphologies were favored. The formation of the nanofibers also depended on the monomer concentration. With the conversion, the morphology of the nanofibers changed from spherical to worm-like due to increasing viscosity [72].

Formation of nanofibers using block copolymers under cylindrical confinement has been reported as well. The two-fluid coaxial spinning technique was used to encapsulate the desired block copolymer as the core with another protective polymer as the shell. Advantages of the two-fluid electrospinning include (a) The shell fluid itself being electro-spinnable serves as a process aid for the block copolymers. Removal of these shells, results in ultrafine block copolymer fibers, (b) smaller diameter fibers are produced due to the independent control of the two fluids that permit wider changes in the diameter of the core fiber, (c) the integrity of the fiber does not get compromised due to the cylindrical confinement of the shell polymer which has a higher glass or melt transition temperature, thus creating a temperature window. TEM images confirm the continuous core–shell nature of the nanofibers. It also shows well-defined structures consisting of concentric layers because of curving of the lamellar phase due to fiber confinement [73].

Polymer/Fe_2_O_3_ hybrid nanofibers have been prepared by processing films of the triblock copolymer polystyrene-*block*-poly(2-cinnamoyloxyethyl methacrylate)-*block*-poly(*tert*-butyl acrylate) (PS-*b*-PCEMA-*b*-PtBA). This preparation involved blocks in thin films getting self-assembled into concentric PCEMA and PtBA cylinders dispersed in a matrix of PS. These cylindrical structures were locked through by photocrosslinking the PCEMA shells. When these shells were dissolved in THF, nanofibers were produced with PtBA cores, PCEMA middle layers, and PS coronas. TEM images revealed PtBA cores that are 20 nm in diameter. Fe_2_O_3_ loading in the caused the nanofiber dispersion to become red. These nanofibers had Fe_2_O_3_ content to be about 0.28 g. TEM images of Fe_2_O_3_ nanofibers revealed fibers around 20 nm, which as the same as the polymer nanofiber [74].

## Conclusion

Nanotechnology has seen many advances in recent years and several synthesis techniques for nanostructures have been developed. These techniques can be mainly classified into top down and bottom-up approaches. Block copolymers have two or more blocks arranged in a defined manner. This arrangement is dependent on factors like Flory–Huggins interaction parameter, degree of polymerization, architectural constraints, and composition. By varying these parameters, it is possible to control the phase behavior and morphology of block copolymers. Such a flexibility enables block copolymers to be used for fabrication of nanostructures as a template or in a solution form. They can also be used to lithographically fabricate nanostructures. One dimensional nanostructures like nanowires, nanorods, nanoribbons, and nanofibers have unique electronic, chemical, optical, magnetic, and mechanical properties due to their nanostructure which enable them to have several applications in different fields of Science. Synthesis of such one-dimensional nanostructures using block copolymers has been discussed in this review. The techniques discussed show the versatility and effortlessness of block copolymers to synthesis these nanostructures.
